# Cytokine Release Syndrome After CAR T-Cell Therapy in a 35-Year-Old Patient With *Pneumocystis jiroveci* Pneumonia and Cytomegalovirus Viremia

**DOI:** 10.1155/carm/6751047

**Published:** 2024-12-16

**Authors:** Kristina A. Helms

**Affiliations:** BMT/Hematology, Mayo Clinic, Rochester, Minnesota, USA

**Keywords:** CAR T-cell, case report, CMV, CRS, infection, PJP

## Abstract

**Background:** The risk of cytokine release syndrome (CRS) in patients with infections prior to chimeric antigen receptor T-cell (CAR T-cell) therapy represents an important and underreported event. Patients with active infections needing prompt CAR T-cell therapy to treat aggressive hematologic malignancies remain a clinical challenge.

**Case Report:** This case describes the clinical course of a 35-year-old male patient with relapsed/refractory T-cell/histiocyte-rich large B-cell lymphoma who received axicabtagene ciloleucel. The patient developed ASTCT Grade II CRS on day +5, necessitating hospital admission and intravenous antibiotics, dexamethasone and tocilizumab. The patient was found to have a *Pneumocystis jirovecii* pneumonia (PJP) infection 3 days prior to CAR T-cell infusion and cytomegalovirus (CMV) viremia 3 days after CAR T-cell infusion. He received TMP-SMX for 21 days to treat PJP and valganciclovir to treat CMV viremia. PET/CT on day +26 demonstrated near resolution of pulmonary nodules and significant partial response of disease according to Deauville criteria.

**Conclusion:** This case highlights the risk of CRS in immunocompromised patients with infections, and presents a unique case of CRS associated with PJP and CMV infections. Although the patient's clinical course was fraught with complications, he achieved a significant partial response to CAR T-cell therapy with the help of a multidisciplinary medical team.

## 1. Background

Chimeric antigen receptor T-cell (CAR T-cell) therapy has revolutionized the treatment landscape of aggressive hematological malignancies since its inception in 2002. Lymphocytes are engineered to produce CARs directed toward tumor cell antigens. Clinical trials and observational studies have shown that CD19 CAR T-cell therapy provides excellent antitumor activity with better progression-free survival (PFS) and overall survival (OS) rates compared with alternate therapies [1]. Unfortunately, CAR T-cell administration can have associated toxicities including cytokine release syndrome (CRS), immune effector cell-associated neurotoxicity syndrome (ICANS), hypogammaglobulinemia, and prolonged cytopenia [2]. As a result of underlying immune system dysregulation, hematologic malignancies, lymphodepleting (LD) chemotherapy, and immunosuppressive treatment for CRS and ICANS, patients who undergo CAR T-cell therapy are predisposed to infections [3].

Several studies found that tumor burden, the intensity of LD chemotherapy, CAR T-cell dose, and thrombocytopenia were risk factors of CRS [4–6]. Infections after CAR T-cell therapy have been described [7], however, little is known about the risk of CRS in patients with infections, partially due to infections representing a major exclusion criteria from large registration trials [8, 9]. In addition, cytomegalovirus (CMV) reactivation in the posttreatment phase has been described but remains an underrecognized complication after CAR T-cell therapy [10].

Generally, infections should be treated prior to CAR T-cell therapy. However, in patients with active infections needing prompt CAR T-cell therapy to treat aggressive disease, the risks of delaying lymphoma-directed therapy need to be evaluated as these risks may be greater than the risks of infectious complications. The relative paucity of reports concerning active infections prior to CAR T-cell therapy necessitates the presentation of distinctive clinical courses that outline the risks of CRS complications with infections.

## 2. Case Report

A 35-year-old male patient presented to the emergency department with a sudden onset of chest tightness, dyspnea, and fever. Medical history was positive for T-cell/histiocyte-rich large B-cell lymphoma for which he received multiple lines of therapy including four cycles of R-CHOP^∗^, two cycles of R-DHAP^∗^, and 2 cycles of R-IGEV^∗^ with progression of disease. He endorsed “poking” chest pain with deep inspiration or coughing at the time of initial consultation at our medical center. The most recent CT of the chest, abdomen, and pelvis showed an enlarging distal periesophageal lymph node along with new pulmonary nodules and increased ground-glass infiltrate in the right upper lung and scattered mosaic attenuation as well as interval enlargement of gastrohepatic ligament and para-aortic lymphadenopathy comparing to previous imaging from two months prior. There was no change in the lymphadenopathy elsewhere including the large right common and external iliac lymph nodes. Staging was Deauville 5. Excisional (inguinal) lymph node biopsy confirmed T-cell/histiocyte-rich B-cell lymphoma.

**Figure 1:**
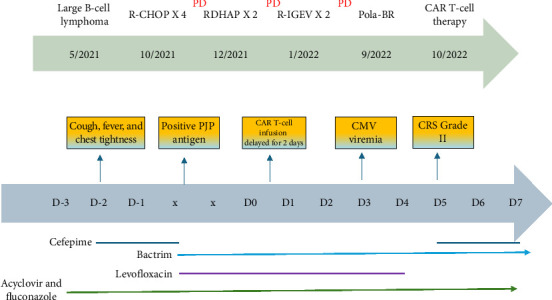
Chronology of events.

The plan was to treat his primary refractory disease with CAR T-cell therapy, specifically axicabtagene ciloleucel (axi-cel). He received bridging chemotherapy with polatuzumab-BR^∗^. He had a mixed response to the Pola-BR. He had an overall partial response of the FDG avid lymphadenopathy above and below the hemidiaphragm, specifically the gastrohepatic nodal mass and right pericaval conglomerate of lymph nodes. Unfortunately, there was an interval increase in the number of FDG avid bilateral pulmonary nodules, hilar, and mediastinal lymph nodes. He completed three days of LD chemotherapy with cyclophosphamide and fludarabine. However, on the day of completion of LD chemotherapy and Day 2 preceding planned CAR T-cell therapy with axi-cel, he presented to the ED with a fever of 39.2°C, chest tightness, and dyspnea (Figure 1).

**Figure 2:**
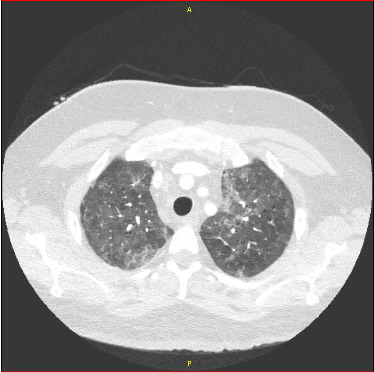
CT chest on the day of admission.

CT angiogram of the chest was performed and pulmonary embolism was ruled out. However, it revealed increasing bilateral diffuse ground-glass opacities with known pulmonary nodules which suggested an infectious, inflammatory or lymphomatous etiology (Figure 2). His laboratory workup revealed neutropenia with an absolute neutrophil count (ANC) of 0.49 × 10(9)/L and a C-reactive protein (CRP) peak of 138.8 mg/L. It is to be noted that he had documented severe neutropenia of ANC ≤ 0.50 × 10(9)/L for six days prior to presenting to the ED. IgG level was 385 mg/dL one week before his hospitalization and he was given immune globulin 10% infusion 30 g at that time. His IgG level was not rechecked during his hospitalization. Pertinent labs from the date of hospital admission to positive *Pneumocystis jirovecii* pneumonia (PJP) antigen are detailed in Table 1.

The nasopharyngeal respiratory viral pathogen profile including SARS-CoV-2 PCR was unrevealing. In addition, he had ongoing campylobacter-associated diarrhea which had been previously diagnosed in the outpatient setting. He was being treated with a seven-day course of azithromycin. During his hospitalization, he was given one dose of ceftriaxone and vancomycin in the ED and then transitioned to intravenous cefepime for empiric treatment of neutropenic fever. He required 1-2 L of supplemental oxygen. CAR-T infusion Day 0 was delayed to ensure active infection was not present or had been adequately treated to avoid the potential for increased complications. The infectious disease team was consulted for further evaluation and management. Further noninvasive workup was obtained including Legionella antigen, methicillin-resistant *staphylococcus aureus* (MRSA) PCR, 1,3-beta glucan serum level, *Pneumocystis jirovecii* PCR, bacterial culture, and SARS-CoV-2 PCR on induced sputum. The patient had a recent COVID-19 infection two months prior to this incident so there was a concern for persistent COVID-19 infection, given his B-cell immunocompromised state.

The pulmonary medicine team was also consulted for further evaluation. Bronchoscopy and bronchoalveolar lavage (BAL) with immunocompromised host (ICH) protocol were considered but not pursued due to the potential proinflammatory state that may be caused by BAL and concern for increasing the risk of CRS with CAR T-cell therapy.

**Figure 3:**
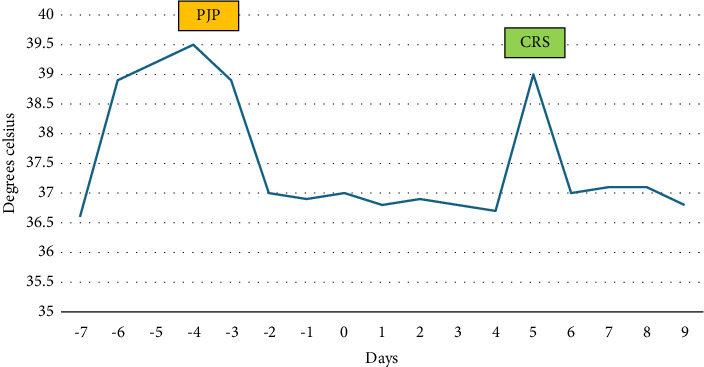
Fever curve.

**Figure 4:**
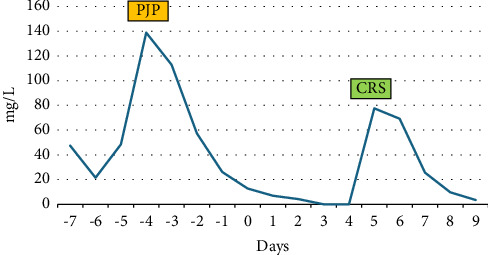
CRP curve.

The 1,3-beta glucan serum was strongly positive at > 500 pg/mL which led to a high index of suspicion for PJP. He was immediately started on trimethoprim–sulfamethoxazole (TMP–SMX) 10 mg/kg. PJP PCR came back positive the next day and he was treated with a 21-day course of TMP–SMX. At this time, intravenous cefepime was discontinued. He was weaned off of supplemental oxygen as well. The rest of the infectious workup came back negative. His fever curve (Figure 3) and CRP levels (Figure 4) improved upon starting PJP-directed therapy. There were detailed discussions between care teams regarding the appropriate timing of CAR-T therapy to give way for antimicrobial therapy to treat active infection with PJP. The risks and benefits of delaying CAR-T therapy were discussed. While CAR-T could have been delayed further, there was a concern that the patient's disease will progress or that the value of LD chemotherapy will be lost. In addition, due to his stable clinical picture, resolution of fevers, and downtrend of CRP, it was decided by hematology, infectious disease, and pulmonology providers to proceed with CAR T-cell infusion, three days after initiation of PJP treatment and six days after completion of LD chemotherapy. He tolerated CAR T-cell infusion well and did not develop any immediate substantial symptoms including fever, CRS, or ICANS. He was started on CRS prophylaxis with dexamethasone for Days +0–2, as well as neutropenic prophylaxis with levofloxacin, fluconazole, and acyclovir per CAR-T protocol. CMV level was drawn per protocol, and he was noted to have a detectable CMV level of 120 IU/mL on Day +3. CMV level remained elevated at 152 IU/mL on Day + 10. Given his increasing viral load, shared decision-making was utilized between the patient, the hematology team, and the infectious disease team to have him start on valganciclovir 900 mg PO BID until there are two consecutive undetected CMV levels one week apart. He was discharged from the hospital on Day +3 after CAR-T infusion and was followed closely in the hospital-based outpatient setting for blood work and evaluation until Day +5 when he met the hospital admission criteria.

**Figure 5:**
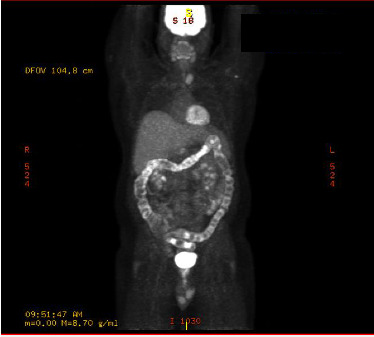
PET/CT on Day +26.

**Figure 6:**
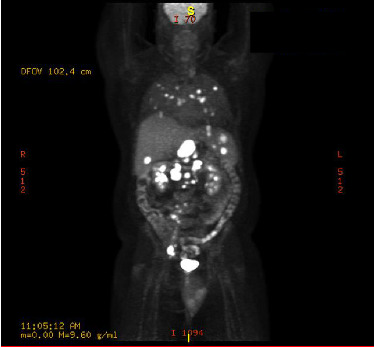
PET/CT two months prior to CAR-T therapy.

On Day +5, he was readmitted for a fever of 39°C and CRS Grade II according to ASTCT consensus grading. He was given one dose of tocilizumab, one dose of intravenous dexamethasone, and a total of 4 L of fluids for hypotension. He was hemodynamically stable with CRS Grade 0 by the evening of Day +5 and was discharged the next day. He was treated with a three-day course of intravenous cefepime and then transitioned back to prophylactic levofloxacin. The infectious evaluation included chest X-ray, CBC, BMP, lactate, blood cultures, urinalysis, and urine culture which were all unremarkable. He was discharged from the hospital on Day +6 and was followed in the hospital-based outpatient for blood work and evaluation on an as-needed basis until Day +25. PET/CT on Day +26 showed near resolution of pulmonary nodules and significant partial response of FDG avid lymphadenopathy with splenic and hepatic involvement (staging was Deauville 4) (Figure 5) compared to PET/CT two months prior to CAR-T therapy (Figure 6).

## 3. Discussion

CAR T-cell therapy is a progressively advancing treatment for blood cancer that can be associated with unique toxicities such as CRS, ICANS, and infections. CRS and infections during the peri-CAR-T period remain a causality dilemma. Studies have shown that there is a significant association between CRS and infections [27] but data are still unclear if CRS precedes an infection or vice versa. In addition, patients who receive CAR T-cell therapy are often cytopenic and immunosuppressed, hence there is a clear risk for infection-related complications.

Possible factors contributing to cytopenias in CAR-T therapy include the number of previous lines of therapy, LD chemotherapy, bridging chemotherapy, underlying malignancy, and baseline marrow reserves [11]. In the registration trials for axi-cel, tisagenlecleucel, and lisocabtagene maraleucel, infection occurred in 12%–55% of patients within the first year [8, 12, 13]. During the first 30 days after CAR T-cell therapy, bacterial infections are the most common with neutropenia as a notable risk factor [14].

Viral infections, on the other hand, are more common after 30 days post-CAR T-cell infusion with lymphopenia and hypogammaglobulinemia as notable risk factors [3]. The majority of these infections were due to respiratory viruses. The incidence and clinical significance of CMV viremia and CMV disease have been increasingly reported after CAR T-cell therapy [10, 15–19]. The interpretation of these studies is limited since routine monitoring of CMV varies among centers. A single-center retrospective study revealed that CMV reactivation (defined as CMV DNA qPCR > 400 IU/mL, *p* = 0.02) and treatment with standard-of-care axi-cel (*p* = 0.02) were significantly associated with an increased risk of death [15]. Well-defined CMV surveillance is necessary to detect reactivation and determine the risks and benefits of CMV prophylaxis and early treatment after CAR-T cell therapy [15].

Fungal infections, though rarer than bacterial and viral infections [20], are linked with significantly poorer prognosis [21]. Important risk factors for fungal infections include duration of neutropenia and lymphopenia and prolonged course of systemic corticosteroid use [3]. A single-center retrospective study showed a low incidence, an 18-month cumulative incidence of 3.8% (95% CI: 1.8–7.8), of invasive fungal disease (IFD) after CD19 CAR-T therapy despite no antifungal prophylaxis [22]. Although antifungal prophylaxis may not be indicated in institutions with low rates of IFD, routine PJP prophylaxis in patients with DLBCL may be considered. A study revealed that PJP was independently associated with lymphopenia, antilymphocyte monoclonal antibodies, high-dose steroid treatment, low albumin, and low body mass index [23]. The association between PJP and rituximab has been described [23, 24]. Therefore, PJP prophylaxis may be considered in patients with DLBCL, especially those who have relapsed/refractory disease with multiple risk factors for PJP such as lymphopenia and treatments with rituximab and high-dose steroids. Although studies have shown that PJP prophylaxis significantly reduces the incidence rate of PJP in DLBCL patients [25–27], there are no standard guidelines for routine PJP prophylaxis in patients with B-cell lymphoma.

As previously discussed, infections should be treated prior to CAR T-cell therapy. However, in patients with active infections needing prompt CAR T-cell therapy to treat aggressive disease, the risks of delaying lymphoma-directed therapy need to be evaluated as these risks may be greater than the risks of infectious complications. However, evidence-based data and clinical experience reports concerning active infections prior to CAR T-cell therapy are lacking. A multidisciplinary medical team is necessary to achieve successful clinical outcomes of these complex patients. Future important avenues for investigation include improved infectious disease screening such as CMV surveillance, standardized monitoring of lymphopenias such as serial CD-4 counts, and individualized strategies for PJP prophylaxis in patients with lymphoma.

## Figures and Tables

**Table 1:** Pertinent labs from the date of hospital admission to positive PJP antigen.

Day of hospitalization	1	2	3	4	5	6 (positive PJP antigen)	7	8
Hemoglobin (g/dL)	13.1	12.4	12.7	13	12.2	11.6	12.5	12.9
Hematocrit (%)	36.3	39.8	40.0	38.4	36.4	35.6	38.2	38.7
Platelets (×10 ^ 9/L)	152	176	154	148	159	144	151	131
WBC (×10 ^ 9/L)	2.3	3.0	1.8	1.7	0.5	0.5	0.8	0.7
Neutrophils (×10 ^ 9/L)	0.83	0.99	1.19	1.03	0.43	0.38	0.70	0.53
Lymphocytes (×10 ^ 9/L)	0.08	0.04	0.03	0.03	< 0.03	0.04	0.06	0.11
Creatinine (mg/dL)	0.82	0.91	0.79	0.76	0.75	0.81	0.88	0.97
BUN (mg/dL)	10	6	7	9	14	18	20	20
eGFR (ml/min/BSA)	> 90	> 90	> 90	> 90	> 90	> 90	> 90	> 90
Ferritin	170	208	351	584	500	498	373	401
CRP (mg/L)	21.5	41.4	138.8	112.9	57.3	26.1	12.8	6.9
LDH (U/L)	264	232	281	265	235	203	202	185
Aspartate aminotransferase (U/L)	18	14	19	16	14	15	16	23
Alanine aminotransferase (U/L)	13	14	12	9	8	13	19	26
Alkaline phosphatase (U/L)	61	71	71	71	73	72	76	83
Total bilirubin (mg/dL)	0.3	0.3	0.4	0.4	0.3	0.2	< 0.2	< 0.2

## Data Availability

The data used to support the findings of this study are available from the corresponding author upon request.

## Nomenclature

ASTCT: American Society for Transplantation and Cellular Therapy
R-CHOP: Rituximab, cyclophosphamide, doxorubicin, vincristine, and prednisone; first-line therapy for non-Hodgkin lymphoma
R-DHAP: Rituximab, dexamethasone, cytarabine, and cisplatin; salvage therapy for relapsed/refractory disease
R-IGEV: Rituximab, ifosfamide, mesna, gemcitabine, vinorelbine, and prednisone; salvage therapy for relapsed/refractory disease
Pola-BR: Polatuzumab, bendamustine, and rituximab; bridging chemotherapy prior to CAR T-cell therapy
Admission criteria: Fever of 38.3 or greater regardless of neutrophil count, unstable vital signs, supportive care for symptom management, and not adequately managed outpatient
